# Longitudinal rate of change in depression symptoms from pre- to post-COVID-19 onset among US mothers

**DOI:** 10.1093/eurpub/ckac129.750

**Published:** 2022-10-25

**Authors:** J Kapp, D Coble, A Kemner, B Hall

**Affiliations:** 1 Health Management and Informatics, University of Missouri School of Medicine, Columbia, USA; 2 Division of Biostatistics, Washington University in St. Louis, Saint Louis, USA; 3 Parents as Teachers National Center, Saint Louis, USA

## Abstract

**Background:**

The extent of the psychological impact of the pandemic is still unfolding. Despite existing literature, most studies lack rigor. We assessed the longitudinal rate of intra-individual change in maternal depression symptoms from before to after COVID-19 onset among US mothers enrolled in a home visiting program with robust adjustment for family contextual factors. We hypothesize that the rate of change in maternal depression symptoms increased after the pandemic onset.

**Methods:**

Eligibility included mothers with ≥1 depression assessment both prior to and after March 16, 2020; thresholds of ≥ 13 on the Edinburgh Postnatal Depression Scale and ≥10 on the Patient Health Questionnaire-9 identified probable depression. We used a generalized linear mixed effects longitudinal model with a random intercept and random slope for time (years) to analyze probable depression (event=‘Yes') pre- and post-COVID. Covariates for model estimation were based on the literature and theory.

**Results:**

Our cohort of 3,431 mothers included 43% non-Hispanic White, 21% non-Hispanic Black, and 31% Hispanic races/ethnicities; 58% from rural/small towns, 18% Spanish-speaking, 63% with one child, median age of 29 and median 2 years follow-up. Households included: 82% low income, 24% low education, 10% insecure housing, 29% single parents, 21% mental illness, 10% substance abuse, and 8% domestic violence. Fourteen percent screened positive for depression pre-COVID, and 10% post-COVID. Depression was significantly higher pre- versus post-COVID, with no significant difference in the rate of change over time. Significant variables (p < 0.05) associated with depression included race/ethnicity, region of the country, number of home visits, mental illness, substance abuse, and domestic violence.

**Conclusions:**

After controlling for family contextual factors, we did not find a significant increase in maternal depression post-COVID-19. Additional research is needed to examine subgroups and the timing of events.

**Key messages:**

• The extent of the psychological impact from the pandemic is still unfolding.

• It is difficult to fully articulate its effects without rigorous, longitudinal research designs.

